# High-resolution chromosomal microarray analysis of early-stage human embryonic stem cells reveals an association between X chromosome instability and skewed X inactivation

**DOI:** 10.1186/2045-3701-4-74

**Published:** 2014-12-02

**Authors:** Yumei Luo, Jieliang Li, Detu Zhu, Yong Fan, Shaoying Li, Xiaofang Sun

**Affiliations:** Key Laboratory for Major Obstetric Diseases of Guangdong Province; Key Laboratory of Reproduction and Genetics of Guangdong Higher Education Institutes, The Third Affiliated Hospital of Guangzhou Medical University, 63 Duobao Road, Liwan District, Guangzhou, Guangdong 510150 People’s Republic of China

**Keywords:** Human embryonic stem cells, Skewed X chromosome inactivation, Chromosomal microarray analysis, Genome instability

## Abstract

X chromosome inactivation (XCI) is a dosage compensation mechanism that silences the majority of genes on one X chromosome in each female cell via a random process. Skewed XCI is relevant to many diseases, but the mechanism leading to it remains unclear. Human embryonic stem cells (hESCs) derived from the inner cell mass (ICM) of blastocyst-stage embryos have provided an excellent model system for understanding XCI initiation and maintenance. Here, we derived hESC lines with random or skewed XCI patterns from poor-quality embryos and investigated the genome-wide copy number variation (CNV) and loss of heterozygosity (LOH) patterns at the early passages of these two groups of hESC lines. It was found that the average size of CNVs on the X chromosomes in the skewed group is twice as much as that in the random group. Moreover, the LOH regions of the skewed group covered the gene locus of either *XIST* or *XACT*, which are master long non-coding RNA (lncRNA) effectors of XCI in human pluripotent stem cells. In conclusion, our work has established an experimentally tractable hESC model for study of skewed XCI and revealed an association between X chromosome instability and skewed XCI.

**Electronic supplementary material:**

The online version of this article (doi:10.1186/2045-3701-4-74) contains supplementary material, which is available to authorized users.

## Introduction

X chromosome inactivation (XCI) is the mechanism by which dosage compensation of the sex chromosome is achieved in females. XCI evolved into a random process in cells from the embryo proper, with either the maternal or paternal X chromosome being inactivated in a random fashion [1]. However, skewed XCI, that is, with only maternal or only paternal X chromosome being inactivated in the whole individual, has been linked to the development of various diseases, including premature ovarian failure, spontaneous miscarriage and some cancers and may induce or deteriorate X-linked genetic diseases, including Rett syndrome, Duchenne muscular dystrophy and fragile X syndrome, via expressing the recessive mutant allele solely [2–7].

The initiation and maintenance of XCI are essentially dependent on long non-coding RNAs (lncRNAs). The most important effector is *XIST* (X-inactive specific transcript), whose gene is mapped on Xq13 (73,040,486-73,072,588 bp) and composes of the X inactivation center (XIC) along with another two RNA genes, *JPX* and *FTX*. In female cells, the *XIST* gene is active on only one of X chromosomes, expressing a large (17 kb), non-coding transcript that coats and silences the chromosome in cis [8]. In human preimplantation embryos, *XIST* is expressed from both paternal and maternal X chromosomes but does not lead to chromosome-wide silencing, indicating a role in XCI initiation [9]. Recently, *XACT* (X-active coating transcript), whose gene is located on chromosome Xq23 (112,983,323-113,235,148 bp) in an unusually large intergenic domain of 1.7 Mb (only 1% of intergenic regions in humans are >1.5 Mb), has been identified as the first lncRNA that coats the active X chromosome specifically in human pluripotent stem cells, indicating a role in the specific kinetics of XCI in humans [8]. However, epigenetic mechanism that is causing or associated with skewed XCI remains unclear.

Previous studies have characterized XCI status in human embryonic stem cells (hESCs) found it an excellent model system to investigate the association between epigenetic alternations and XCI [10, 11]. It has been reported that XCI variations already exist in the early passages (passage 5 to 15) of hESCs, which may be a consequence of *in vitro* culture selection during the derivation process [12, 13]. Single nucleotide polymorphism (SNP) analysis indicated hESCs at early passages had relative genome stability; however, the instability becomes stronger with the increase in passage number (passage >20) [14]. Therefore, it would be better to evaluate the XCI status of hESCs at early stages that have been minimally exposed to culture effects.

Chromosomal microarray analysis (CMA) has emerged as a new high-throughput technique to investigate the genome-wide CNV and loss of heterozygosity (LOH) patterns in hESCs. CNV is a major form of genome structural variation that relative large regions (1 kb to several Mbs in size) of certain chromosome have been deleted (loss) or duplicated (gain). LOH is another major form of variations that a gross region of the chromosome loses one parental copy due to deletion or uniparental disomy. Thus, an increase of CNV and LOH represents higher genome instability. In previous studies, CMA of human pluripotent stem cell lines have identified a CN gain of chromosome 20q11.21 shared in >20% of hESC lines and 18% of human induced pluripotent stem cells, and the cells containing this amplicon have a higher population doubling rate, which is attributable to enhanced resistance to apoptosis [15–18]. BCL2L1, a gene within this common amplicon, is later demonstrated to be a major effector for driving culture adaptation of hESCs [19]. Hence, CMA is a powerful tool to identify genome loci associated to specific traits in hESCs.

In this study, we established 9 hESC lines from poor-quality embryos to generate an experimentally tractable human cellular model to investigate random versus skewed XCI patterns. We classified 3 cell lines with random XCI pattern and another 3 lines with skewed XCI pattern, and compared their genome-wide CNV and LOH patterns via CMA at early passages. Our data showed that CNVs on the X chromosomes of the skewed group were twice more than those of the random group. Moreover, the LOH regions of the skewed group covered either the *XIST* or the *XACT* locus. In conclusion, our work indicated an association between increased X chromosome instability and skewed XCI, and we speculated that LOH in either the *XIST* or the *XACT* locus is a factor that influences XCI patterns.

## Materials and methods

### Deviation and characterization of hESC lines

This study had obtained the approval of the Ethics Committee of The Third Affiliated Hospital of Guangzhou Medical University. Patients were enrolled at the Third Affiliated Hospital of Guangzhou Medical University (Guangzhou, China) and had signed their names by Chinese on written informed consent, agreeing that their abandoned embryos to be used for stem cell research purpose. Poor-quality embryos were cultured in a modified medium for 7 days, as described Fan *et al.* [20]. Only grade 3 and grade 4 embryos were selected. The inner cell mass (ICM) was isolated by mechanical means. All ICMs were plated on mitomycin C-treated murine embryonic fibroblast feeder layers for further culture. After 8–9 days of culture, the colonies derived from the ICM were mechanically dispersed into 2–3 small clumps using a micropipette. The ICM clumps were then transferred to a fresh feeder layer. These cells were again mechanically dissociated during the initial 5 passages. The cells were routinely passed every 4–5 days, and the medium was changed every day. All the cells lines were evaluated for pluripotent potential via in vitro and in vivo differentiation analyses, as previously described by Liu and Sun [21]. All the cell lines were passaged until P8 to P10 and harvested for CMA.

### DNA extraction and purification

Whole-genome DNA was extracted with the QiAamp DNA Blood Mini Kit according to the manufacturer’s instructions (QIAGEN, Germany). DNA was purified with ammonium acetate and ethanol. The concentration and quality of the DNA samples were determined using a spectrophotometer (Nanodrop 2000, Thermo Scientific, USA) and 1% agarose gel electrophoresis with λDNA as a control. A 200 ng sample of DNA was used for the bisulfite reaction. Complete bisulfite conversion and the cleanup of converted DNA was processed using the EpiTect Bisulfite Kit (QIAGEN).

### *HUMARA*gene heterozygosity and XCI assays

The human androgen-receptor (*HUMARA*) gene has a highly polymorphic trinucleotide short tandem repeat (STR) in the first exon, which can be utilized to differentiate heterozygous X chromosomes by AFLP assays. The CpG sites (including HpaII and HhaI sites) <100 bp away from this STR are methylated on inactive X chromosomes but remain unmethylated on active X chromosomes, which can be utilized to detect the XCI pattern by methylation-specific PCR assays. The PCR primer sets for analysis of *HUMARA* gene heterozygosity and methylation pattern are designed based on Liu and Sun [21], whose sequences can be found in Additional file 1: Table S1. The method is more illustrated in Additional file 2: Figure S1. The amplification system for extracted DNA samples was as follows: 10 × RT-PCR Buffer, 1.5 μl; 25 mM MgCl_2_, 0.9 μl; dNTP Mix (10 mM), 1.5 μl; Primer-M/U (10 pmol/μl) F/R, 0.2 μl; Taq Golden Enzyme, 0.1 μl; bisulfite-treated DNA, 1.5 μl; RNase-free water, 9.1 μl. The reaction was performed under the following conditions: 95°C for 12 min, followed by 40 cycles of 30 s at 94°C, 30 s at 55°C, 30 s at 72°C and extension for 7 min at 72°C. A Genetic analyzer 3500 was used for fragment analysis, and the data were then analyzed by Genemapper software (Applied Biosystems, CA, USA).

### Chromosomal microarray analysis

We used high-resolution genotyping microarray Affymetrix CytoScan 750 K Array (Affymetrix, Santa Clara, CA, USA) according to the manufacturer’s instructions. 250 ng undegraded DNA of each sample was used for the array. The expected size of the PCR products was 150–2000 bp, with a concentration of over 300 ng/μl and OD260/280 and OD260/230 values of 1.8-2.0 and 2.0-2.3, respectively. The size of fragmentation was expected to be 25–125 bp. Hybridization using a GeneChip® Hybridization Oven 645 (Affymetrix) was followed by washing using the GeneChip® Fluidics Station 450 (Affymetrix) and scanning using the GeneChip®Scanner 3000 7G (Affymetrix).

The raw data obtained were then analyzed by Chromosome Analysis Suite 2.0 (CHAS2.0) software (Affymetrix) to figure out the genome-wide CNV and LOH pattern based on the Hidden Markov Model. The parameter setting was: CNV size from 50 kb to 3 Mb, marker count >50 and LOH >100 kb.

### Statistical analysis

All data are represented as mean ± s.d. The statistical significance of differences was determined by Student’s *t*-Test. A P value of <0.05 was considered to be statistically significant.

## Results

### Derivation and characterization of hESC lines

**Figure 1:**
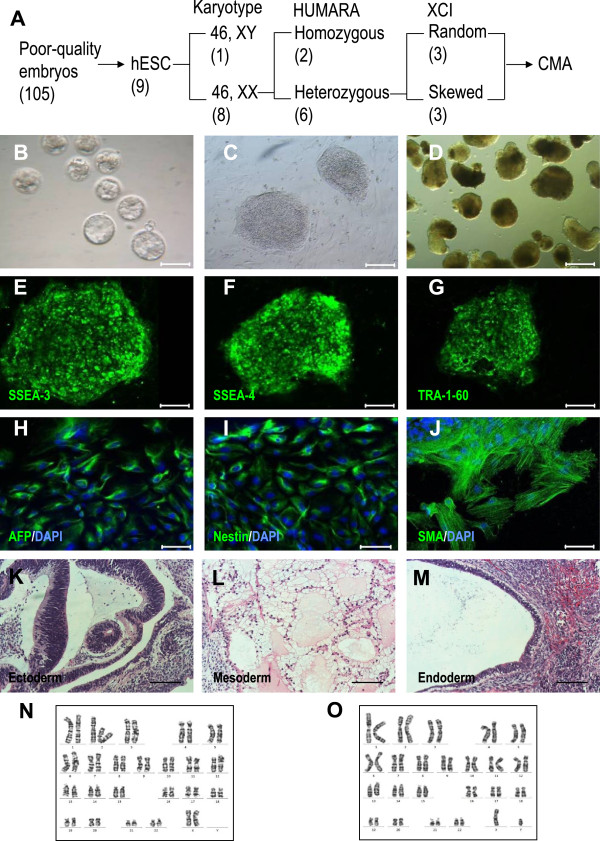
**Derivation and characterization of hESC lines. (A)** Schematic diagram to present the procedure to establish a hESC model from poor-quality embryos for study of skewed XCI. The numbers in brackets indicate the number of cell lines in each group. **(B)** Representative poor-quality embryo culture in modified medium. **(C)** Colony morphology under an inverted microscope. **(D)** EB formation at day 7 when plated in suspension dishes. **(E-G)** Immunostaining analysis of hESCs for anti-SSEA-3 (green), anti-SSEA-4 (green) and anti-TRA-1-60 (green). **(H-J)** Immunostaining analysis for in vitro differentiation into cell types representative of the three embryonic germ layers, including alpha-fetoprotein (AFP, endoderm), nestin (ectoderm) and smooth muscle actin (SMA, mesoderm). **(K-M)** Histological examination of in vivo differentiation showing that the teratomas contained tissues of the three embryonic germ layers, including neural-tube like cells (ectoderm), fat cells (mesoderm) and glandular tissue (endoderm). **(N, O)** Normal karyotypes of 46, XX and 46, XY. Bars in **(B-M)**: 100 μm.

Previously we have developed a protocol to derive normal hESC lines from poor-quality embryos [20]. For the present study, our laboratory received a total of 105 poor-quality embryos cultured in a modified medium. Blastocysts formed at day 8 to day 10 (Figure 1B). Then, 21 ICMs were isolated from the blastocysts and plated onto feeder layers, 14 of them attached, and finally 9 cell lines grew out with a typical hESC morphology (Figure 1C). All cell lines expressed hESC markers such as SSEA-3, SSEA-4 and TRA-1-60 (Figure 1E-G). Each cell line was able to form embryonic bodies (EBs) (Figure 1D) and expressed a-fetoprotein (AFP, an endoderm marker), nestin (an ectoderm marker) and smooth muscle actin (SMA, a mesoderm marker) (Figure 1H-J). Teratomas were observed at 4–10 weeks after inoculation into SCID mice. Histological examination revealed that the teratomas contained the tissues of the three embryonic germ layers, including neural tube-like cells (ectoderm), fat cells (mesoderm) and glandular tissue (endoderm) (Figure 1K-M). After characterization of the 9 hES cell lines, we named the 8 hESC lines with the normal karyotype of 46, XX as hES-20, hES-25, hES-26, hES-27, hES-33, hES-34, hES-35 and hES-39 (Figure 1N); the cell line hES-12 had a normal karyotype of 46, XY (Figure 1O).

### Heterozygosity of *HUMARA*gene in hESC lines

**Figure 2:**
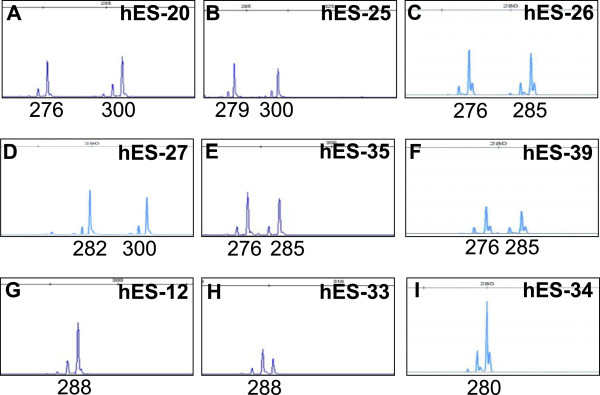
**Heterozygosity of the HUMARA gene in hESC lines.** The polymorphism patterns of the HUMARA gene in the hESC lines show that **(A-F)** 6 cell lines have two different HUMARA alleles, indicating that they are heterozygous at the HUMARA locus: hES-20, hES-25, hES-26, hES-27, hES-35 and hES-39; **(G-I)** the other 3 cell lines have only one HUMARA allele, indicating that they are homozygous at the HUMARA locus: hES-12, hES-33 and hES-34. X-axis indicates the size (bp) of DNA product.

To investigate random and skewed XCI patterns, only heterozygotes can be used to distinguish the maternal and paternal alleles based on polymorphism of the trinucleotide repeat element at the *HUMARA* locus [22]. Therefore, we harvested the 8 female hESC lines and the male hES-12 line, serving as a reference for ‘homozygote’, at passage 8 to 10 and extracted DNA to analyze the polymorphism patterns of the *HUMARA* gene. 6 out of the 9 investigated cell lines were heterozygous at the *HUMARA* locus: hES-20 P9, hES-25 P8, hES-26 P9, hES-27 P10, hES-35 P9 and hES-39 P10 (Figure 2A-F). 3 cell lines were homozygous at the *HUMARA* locus: hES-12 P8, hES-33 P8 and hES-34 P9 (Figure 2G-I). Therefore, the 6 heterozygous cell lines were further analyzed for random or skewed XCI patterns.

### Different XCI patterns in hESC lines

**Figure 3:**
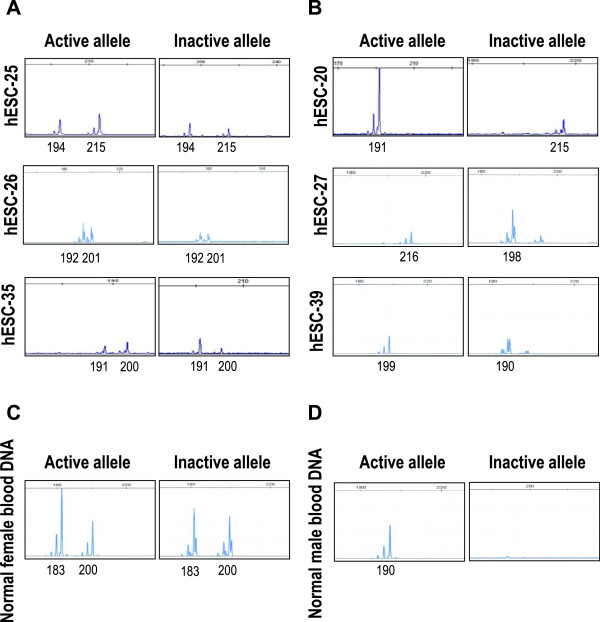
**Different XCI patterns in heterozygous hESC lines. (A)** shows random XCI in hES-25, hES-26 and hES-35 where two active and two inactive alleles of the HUMARA gene are detected. The peak area ratios between the two active or inactive alleles are 50:50, 64:36 and 60:40 respectively. **(B)** shows skewed XCI of hES-20, hES-27 and hES-39 where only one active allele and another different inactive allele of the HUMARA gene are detected. **(C)** shows normal female blood DNA as a control for random XCI (two active and two inactive alleles, 183 and 200 bp, respectively), and **(D)** shows normal male blood DNA with only a 190 bp active allele and no inactive allele detected. X-axis indicates the size (bp) of DNA product.

At early undifferentiated passages, all of the 6 hESC lines had both active and inactive X chromosome alleles. In XCI pattern assays, 3 lines (hES-25 P8, hES-26 P9 and hES-35 P9) had a random XCI pattern, possessing two active and two inactive alleles. The peak area ratios between the two active or inactive alleles were almost 50:50, 64:36 and 60:40 respectively (Figure 3A). The other 3 cell lines (hES-20 P9, hES-27 P10 and hES-39 P10) had extremely skewed XCI patterns (only one active and one inactive allele): only the 191 bp active allele and the 215 bp inactive allele were in hES-20 P12; only the 216 bp active allele and the 198 bp inactive alleles were in hES-27 P10; only the 199 bp active allele and the 190 bp inactive alleles were in hES-39 P10 (Figure 3B). Blood DNA from a normal female who had a random XCI pattern was used as a positive control (Figure 3C), and blood DNA from a normal male who had one active X chromosome and lacked an inactive X chromosome was used as a negative control (Figure 3D). Thus, we have classified 3 cell lines showing random XCI pattern and 3 others showing skewed XCI pattern for further CMA.

### CNV status in hESCs

**Figure 4:**
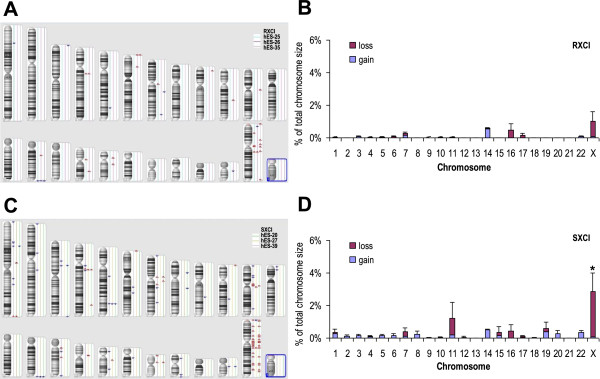
**CNV status in hESC lines. (A)** The genome-wide CNV status of the random XCI hESC group (hES-25 P8, hES-26 P9 and hES-35 P9). **(B)** Average chromosomal distribution of 50 kb-3 MB size CNVs in the random XCI hESC group. **(C)** The genome-wide CNV status of the skewed XCI hESC group (hES-20 P9, hES-27 P10 and hES-39 P10). **(D)** Average chromosomal distribution of 50 kb-3 MB size CNVs in the skewed XCI hESC group. Blue marks indicate regions of copy number gains. Red marks indicate regions of copy number loss. Only CNVs with a size from 50 kb to 3 Mb are counted. RXCI, random X chromosome inactivation. SXCI, skewed X chromosome inactivation. Error bars represent SD. *P <0.05 versus RSCI group.

Genome-wide CNVs were identified with a size range from 50 kb to 3 Mb in the random XCI hESC group (hES-25 P8, hES-26 P9 and hES-35 P9) and the skewed XCI hESC group (hES-20 P9, hES-27 P10 and hES-39 P10) by Affymetrix CytoScan 750 K Arrays (Figure 4A,C). Both groups showed higher CN losses than CN gains and enriched CNVs on the X chromosome (Figure 4B,D). However, there were more CNVs in the skewed group than in the random group. In the random group, the average total percent of CNVs in the genome by size was 0.13%; while in the skewed group, it was 0.38%. Particularly, the average percentage of CNVs in the X chromosome by size was 1.03% in the random group, but it was up to 2.88% in the skewed group, which was twice higher than that of the random group (Figure 4B,D). These results indicated that the skewed group had a high genomic instability, especially in the X chromosome, than the random group.

### LOH analysis in hESCs

**Figure 5:**
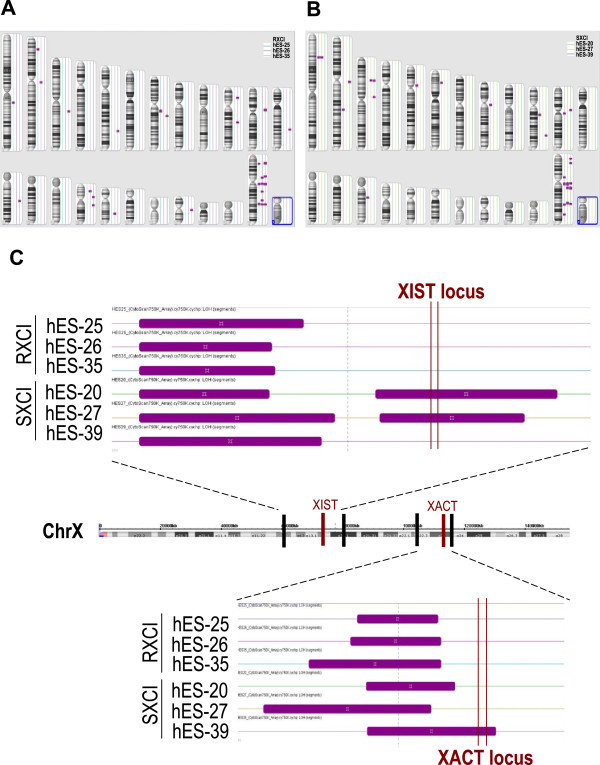
**LOH status in hESC lines. (A)** The genome-wide LOH status of the random XCI hESC group. **(B)** The genome-wide LOH status of the skewed XCI hESC group. **(C)** Enlarged diagram showing the LOH status around the *XIST* locus and the *XACT* locus. Purple bars indicate regions of LOH. Only LOHs with a size larger than 100 kb are counted.

LOH was identified with a size larger than 100 kb. In both random and skewed groups, LOH was mainly associated with the X chromosome (Figure 5A,B) and large regions of LOH (all were larger than 3 Mb) were commonly found at the 5’ upstream of the *XIST* locus at Xq13.2 and the *XACT* locus at Xq23 (Figure 5C). The difference was, in the skewed XCI group, the LOH regions of hES-20 and hES-27 covered the *XIST* locus and in hES-39, the LOH region included the *XACT* locus (Figure 5C). *XIST* is a master effector of the inactive X chromosome and *XACT* is a newly discovered long non-coding RNA that coats the active X chromosome specifically in human pluripotent stem cells. Hence, the LOHs in these loci might correlate with the skewed XCI pattern.

## Discussion

X-chromosome inactivation (XCI) results in the silencing of most genes on one X chromosome, yielding mono-allelic expression in individual female cells; while random XCI results in the expression of both alleles in most females [13]. In female embryos, XCI is present as early as the 8-cell stage [23]. In undifferentiated female hESCs at intermediate passages, the XCI state is variable: Class I hESCs are at pre-XCI state with two active X chromosome, and initiate XCI upon differentiation; Class II hESCs are at post-XCI state with one inactive X chromosome; Class III hESCs also retain an inactive X chromosome despite absence of XCI markers [24]. Class II hESCs are found in most cases [24]. Dvash et al. found that hESC lines at early passages already exhibit various XCI states, including the skewed XCI pattern, and suggested that it might reflect innate heterogeneity of the original cells found in the ICM of the human embryo [12]. Hence, the generation of hESCs provides an invaluable tool to study the epigenetic mechanism of XCI initiation during embryogenesis. In the present study, we isolated the ICM from poor-quality embryos and established 8 female hESC lines used as a model system to understand XCI initiation and maintenance. All of the hESC lines exhibited pluripotent gene expression, normal chromosome G-banding and teratoma formation in vivo.

The data available thus far on XCI patterns in hESCs are diverse. Different XCI patterns in undifferentiated female hESCs have been reported in different cell lines [25, 26]. To examine XCI skewing, the presence of a polymorphism was required to distinguish the two X chromosomes and to determine which X chromosome is inactive [22]. Therefore, we initially analyzed the heterozygosity of the X-linked *HUMARA* gene. Because hESCs are derived from human fertilized oocytes, the heterozygosity between adult females and female hESC lines should be the same [27]. Our data showed that 75% (6 out of 8) female hESCs lines were heterozygous at the *HUMARA* locus, which is a similar rate to adult females [27]. Therefore, only 6 heterozygous cell lines were further analyzed for random or skewed XCI patterns.

Although the hESC lines were cultured under the same condition, both random and skewed XCI patterns could be found in them [21]. In our study, we harvested hESC lines from passage 8 to 10 for XCI pattern assays. If random XCI occurs in hESCs, there should be two active alleles and two inactive alleles, and the peak area ratio of both the active and inactive alleles should be between 50:50 to 80:20 [21]. Skewed XCI is arbitrarily defined as more than 80% of cells showing a preferential inactivation of the same X chromosome [28]. Our data demonstrated that 3 lines (hES-25 P8, hES-26 P9 and hES-35 P9) had a random XCI pattern, possessing two active and two inactive alleles. The peak area ratios between the two active or inactive alleles were almost 50:50, 64:36 and 60:40. Meanwhile, the other 3 lines (hES-20 P9, hES-27 P10 and hES-39 P10) exhibited extremely skewed XCI patterns (only one active and one inactive allele). Therefore, we propose that culture condition may not be a key influencing factor for adaptive selection of skewed XCI.

CMA is an emerging high-throughput technique to profile the genome-wide CNV and LOH patterns in hESC lines. Our Affymetrix CytoScan 750 K Array data showed that the CNV patterns were different between the skewed XCI hESC group and the random group. In the skewed group, the average percent of CN gain by size was 0.14% of the whole genome and 0.07% of the X chromosome; the average CN loss was 0.14% of the whole genome and 2.81% of the X chromosome. In the random group, the average percent of CN gain by size was 0.04% of the whole genome and 0.08% of the X chromosome; the average CN loss was 0.08% of the whole genome and 0.95% of the X chromosome. In both groups, CN losses were more than gains; meanwhile, more CNVs were found on the X chromosome. These data revealed that the skewed group had higher X chromosome instability than the random group.

*XIST* is a noteworthy non-coding transcript in that, in addition to silencing an entire chromosome, it is the only lncRNA described thus far to widely coat the chromosome from which it is expressed [8]. This coating induces nuclear reorganization and recruitment of histone-modifying complexes that are important for the initiation and maintenance of XCI [29]. The *XACT* gene is located on chromosome Xq23 between the protein-coding genes *AMOT* and *HTR2C* in an unusually large intergenic region [8]. It was the first lncRNA identified to coat the active X chromosome specifically in human pluripotent stem cells. *XACT* might not be conserved in mice, and its function might be related to the specific kinetics of XCI that were recently described in humans [8]. Vallot et al. speculate that *XACT* is one of the rapidly evolving lncRNAs that involve in the species-specific regulation of XCI initiation in human, just like *Tsix* in mice [8]. In our study comparing the skewed XCI hESC group and random group, LOH was mainly associated with the X chromosome. In the skewed group, the LOHs covered the *XIST* locus at Xq13 in hES-20 and hES-27 and the *XACT* locus at Xq23 in hES-39, which was not found in the random group. We speculate that LOH in either *XIST* or *XACT* locus is a factor that impacts skewed XCI pattern.

## Conclusion

In conclusion, the data presented here highlight the diverse XCI status in early-passage hESCs and, most importantly, increased instability on X chromosome and LOHs covering either *XIST* or *XACT* gene locus in hESCs with skewed XCI. More in-depth work is needed to characterize the association between X chromosome instability and skewed XCI in these cells.

## Electronic supplementary material

Additional file 1: Table S1: Primer sets used in this study. (PDF 7 KB)

Additional file 2: Figure S1: Illustration of positions of CpG sites, polymorphic CAG trinucleotide repeat and primer sets. (PDF 113 KB)

## Abbreviations

AFP: A-fetoprotein
CMA: Chromosomal microarray analysis
CNV: Copy number variation
EB: Embryonic body
hESC: Human embryonic stem cells
*HUMARA*: Human androgen-receptor gene
ICM: Inner cell mass
lncRNA: Long non-coding RNA
LOH: Loss of heterozygosity
SMA: Smooth muscle actin
SNP: Single nucleotide polymorphism
STR: Short tandem repeat
*XACT*: X-active coating transcript
XCI: X chromosome inactivation
XIC: X inactivation center
*XIST*: X-inactive specific transcript.
