# Supplementary material for: High-resolution chromosomal microarray analysis of early-stage human embryonic stem cells reveals an association between X chromosome instability and skewed X inactivation
Source: Cell Biosci. 2014 Dec 2;4:74. doi: 10.1186/2045-3701-4-74 (PMC4265433; doi:10.1186/2045-3701-4-74)
Supplement: Supplementary file 1 — Additional file 1: Table S1: Primer sets used in this study. (PDF 7 KB) [file 13578_2014_195_MOESM1_ESM.pdf]

Table S1 Primer sets used in this study

Name	Primer sequence (5' to 3')	Size (bp)
HUMARA	F:GCTGTGAAGGTTGCTGTTCCTCAT	258-303*
	R:TCCAGAATCTGTTCCAGAGCGTGC	
HUMARA-M	F:GCGAGCGTAGTATTTTTCGGC	177-221*
	R:AACCAAATAACCTATAAAACCTCTACG	
HUMARA-U	F:GTTGTGAGTGTAGTATTTTTTGGT	177-221*
	R:CAAATAACCTATAAAACCTCTACA	

F: forward primer;

R: reverse primer;

* indicates polymorphism patterns of the *HUMARA* gene.
