# Supplementary material for: High-resolution chromosomal microarray analysis of early-stage human embryonic stem cells reveals an association between X chromosome instability and skewed X inactivation
Source: Cell Biosci. 2014 Dec 2;4:74. doi: 10.1186/2045-3701-4-74 (PMC4265433; doi:10.1186/2045-3701-4-74)
Supplement: Supplementary file 2 — Additional file 2: Figure S1: Illustration of positions of CpG sites, polymorphic CAG trinucleotide repeat and primer sets. (PDF 113 KB) [file 13578_2014_195_MOESM2_ESM.pdf]

```
241 TTCCAGAGCGTGCGCGAAGTGATCCAGAACCCGGGCCCCAGGCACCCAGAGGCCGCGAGC
    ||::||||++||++++||||||::||||::++||::::|||:|:::|||||:++++||+
241 TTTTAGAGCGTGCGCGAAGTGATTTAGAATTCGGGTTTTAGGTATTTAGAGGTCGCGAGC
                                                      M>>>>>>
                                                   U>>>>>>>>>>

301 GCAGCACCTCCCGGCGCCAGTTTGCTGCTGCAGCAGCAGCAGCAGCAGCAGCAGCAGCAG
    +:||:|::|::++|++::||||||:||:||:||:||:||:||:||:||:||:||:||:||
301 GTAGTATTTTTCGGCGTTAGTTTGTTGTTGTAGTAGTAGTAGTAGTAGTAGTAGTAGTAG
    >>>>>>>>>>>>>>>
    >>>>>>>>>>>>>>>

361 CAGCAGCAGCAGCAGCAGCAGCAGCAGCAGCAAGAGACTAGCCCCAGGCAGCAGCAGCAG
    :||:||:||:||:||:||:||:||:||:||:||||||:|||::::|||:||:||:||:||
361 TAGTAGTAGTAGTAGTAGTAGTAGTAGTAGTAAGAGATTAGTTTTAGGTAGTAGTAGTAG

421 CAGCAGGGTGAGGATGGTTCTCCCCAAGCCCATCGTAGAGGCCCCACAGGCTACCTGGTC
    :||:|||||||||||||||:|::::|||:::||++||||||::::|:|||:||::||||:
421 TAGTAGGGTGAGGATGGTTTTTTTTAAGTTTATCGTAGAGGTTTTATAGGTTATTTGGTT
                                     M<<<<<<<<<<<<<<<<<<<<<<<<<<
                                     U<<<<<<<<<<<<<<<<<<<<<<<
```

Figure S1. Illustration of positions of CpG sites, polymorphic CAG trinucleotide repeat and primer sets. The upper row notes original sequence of *HUMARA* gene, and the lower row shows bisulfite modified sequence of *HUMARA* gene (For display, assume all CpG sites are methylated). The number in the left indicates the genomic position, i.e., the location of the 5' nucleotide of the primer in relation to the first base of the sequence in the following reference and Genbank accession number: HUMARA, M35844 (Tilley, et al., 1989). The sequences marked blue are the position where the primers bind, and the sequences marked red indicate the highly polymorphic CAG trinucleotide repeat. The other markers are illustrated as follows:

```
++        CpG sites
::::      Non-CpG 'C' converted to 'T'
M >>>>>>  Forward methylated-specific primer
M <<<<<<  Reverse methylated-specific primer
U >>>>>>  Forward unmethylated-specific primer
U <<<<<<  Reverse unmethylated-specific primer
```
